# Preliminary Study on Continuous Recognition of Elbow Flexion/Extension Using sEMG Signals for Bilateral Rehabilitation

**DOI:** 10.3390/s16101739

**Published:** 2016-10-19

**Authors:** Zhibin Song, Songyuan Zhang

**Affiliations:** 1 Key Laboratory of Mechanism Theory and Equipment Design of the Ministry of Education, Tianjin University, Tianjin 300354, China; 2 State Key Laboratory of Robotics and System, Harbin Institute of Technology, Harbin 150080, China; zhangsy@hit.edu.cn; 3 Department of Intelligent Mechanical Systems Engineering, Kagawa University, Kagawa Prefecture 761-0396, Japan

**Keywords:** sEMG, continuous recognition, rehabilitation robotics

## Abstract

Surface electromyography (sEMG) signals are closely related to the activation of human muscles and the motion of the human body, which can be used to estimate the dynamics of human limbs in the rehabilitation field. They also have the potential to be used in the application of bilateral rehabilitation, where hemiplegic patients can train their affected limbs following the motion of unaffected limbs via some rehabilitation devices. Traditional methods to process the sEMG focused on motion pattern recognition, namely, discrete patterns, which are not satisfactory for use in bilateral rehabilitation. In order to overcome this problem, in this paper, we built a relationship between sEMG signals and human motion in elbow flexion and extension on the sagittal plane. During the conducted experiments, four participants were required to perform elbow flexion and extension on the sagittal plane smoothly with only an inertia sensor in their hands, where forearm dynamics were not considered. In these circumstances, sEMG signals were weak compared to those with heavy loads or high acceleration. The contrastive experimental results show that continuous motion can also be obtained within an acceptable precision range.

## 1. Introduction

Stroke is one of the most harmful diseases which results in adults losing their ability to read, speak, understand and move. Patients may even lose their lives. Based on reports from seven countries (the US, France, Germany, Italy, Spain, the UK and Japan), stroke occurs in an average of 214 out of 100,000 people per year, with the incidence rate growing annually by 1.9% due to an aging population [1]. With the increase of the aging population, new rehabilitation technology is required more and more urgently, especially using robotics in home rehabilitation. Compared to traditional rehabilitation strategies, robot-mediated rehabilitation has been widely considered as a promising method to improve patient access to therapy [2]. There are several rehabilitation strategies used widely in robot-mediated rehabilitation, including passive training, active training and active-resistce training. On the other hand, bilateral rehabilitation is also a very promising strategy because it is suitable for hemiparesis in home rehabilitation. Over half of stroke patients suffer from hemiparesis, which affects one side of the patient’s body while the other side is unaffected. Bilateral rehabilitation based on robotic systems appeared several years ago [3]. One of the typical systems using bilateral rehabilitation for upper limbs is a Mirror Image Movement Enabler (MIME), which adopted an industry robot (PUMA 560) that applies forces to the paretic limb [4]. In this system, to acquire the motion of the unaffected limb, a forearm splint was used to fix the unaffected limb, and its motion can be detected by the sensors installed on the splint. It is a constraint for unaffected limbs. In this paper, we proposed a method to detect the motion of unaffected limbs without a constraint, which is to adapt surface electromyography (sEMG) signals [5]. Moreover, the sEMG signals can be detected by a wireless communication device [6]. On the other hand, sEMG reflects the activation of muscle and human motion, not only including the movement of human limbs but also including the trend of motion that cannot be detected by other sensors directly.

Generally speaking, there are two main parts to process sEMG signals according to previous literature: feature extraction and feature classification. Moreover, from the perspective of processing, the method of feature extraction can be grouped into three types: time domain, frequency domain and time–frequency domain [7]. The methods of time domain mainly include Integrated EMG (IEMG), Mean Absolute Value (MAV), and so on [7]. The methods of frequency domain mainly include auto-regressive coefficients, frequency median, and so on [8]. The methods of time–frequency domain were developed based on in frequency domain and include Wavelet Transform (WT) and Wavelet Packet Transform (WPT) [9,10,11,12,13]. For the process of feature classification, artificial neural networks were used widely because they are good at dealing with nonlinear problems. On the other hand, a fuzzy logic classifier and support vector machines [14] are also effective methods to deal with the classification of features. In this paper, WPT is chosen as the basic method of feature extraction because of its predominant characteristics in the filter and feature extraction of signals.

In the past several decades, many researchers have studied motion pattern recognition based on sEMG signals, such as in prosthesis. However, much of the research has focused on the recognition of motion patterns, namely, discrete patterns. Continuous motion recognition was merely mentioned. On the other hand, most motion pattern recognitions focused on some special motions that can induce high activation of muscle and strong sEMG signals, such as hand grasping, so that they can obtain high recognition accuracy. In this research, we focused on the recognition of human elbow flexion and extension on the sagittal plane for bilateral rehabilitation with only one electrode attached to the bicep muscle. The subjects were required to perform this motion in the sagittal plane without any load on their upper limbs, except for an inertia sensor (MTx) (30 g). It is more difficult to build the relationship between the sEMG signals and human motion. For bilateral rehabilitation, continuous motion recognition is necessary in real-time. In our previous research, we had developed the upper limb rehabilitation robotics and conducted some experiments for processing the sEMG signals with healthy subjects [15,16,17,18]. In this paper, we first extracted four motion patterns for elbow flexion and extension in the sagittal plane, and compared differences of results between the proposed weighted peak method and the improved version. At last, the continuous motion for elbow flexion and extension was implemented based on two proposed methods.

## 2. Materials and Methods

**Figure 1:**
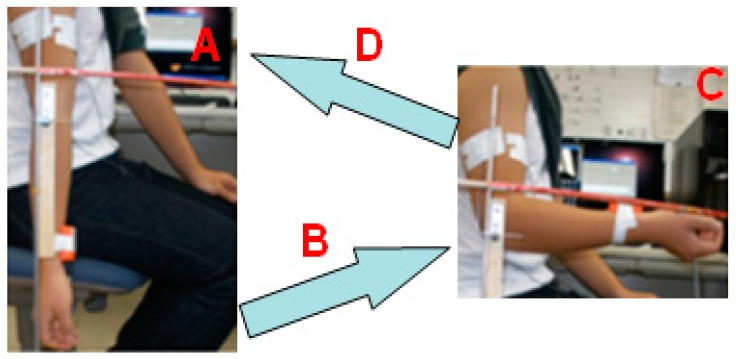
Four motion patterns to be recognized.

To build the mapping between the sEMG signals and elbow flexion and extension on the sagittal plane when the motion is performed very smoothly, so that the dynamic efficacy can be omitted, the statics are analyzed, which are easier and simpler than dynamics. It is not difficult to notice that there are two main forces involved in this motion: gravity and muscle extraction force of the biceps. Thus, it is possible to acquire and process the sEMG signals from the biceps to find the relationship between elbow flexion and extension. The most important phase is to extract the features. In the case of discrete recognition, acquired features are classified into several groups, so the recognition results would be correct as long as the features are classified into the right groups. However, in the case of continuous recognition, the features are not classified into certain groups, but mapped into the quantitative values, such as position, torque, and so on. Therefore, more discriminative features are required for continuous recognition than those for discrete recognition. To evaluate our proposed sEMG processing method, four discrete patterns were classified during elbow flexion/extension, with the upper arm remaining static and perpendicular to the ground, with the forearm hanging down (A), flexion towards horizontal plane (B), lifting in the horizontal plane (C), and extension back to the vertical plane (D) (Figure 1). To ensure that the actions of A and C can be performed correctly, an orthogonal framework was made to indicate the horizontal and vertical plane. During elbow flexion and extension, the upper arm was fixed statically and the forearm was required to move very slowly, according to the four aforementioned motion patterns, so that the motion can be assumed to be in equilibrium.

### 2.1. SEMG Signal Acquisition and Experiments

**Figure 2:**
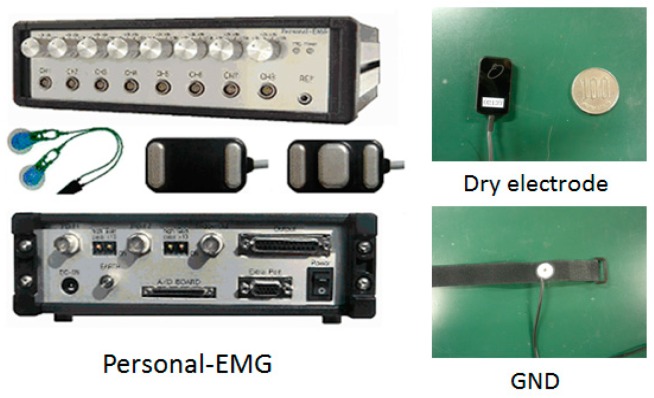
Experimental setup of surface electromyography (sEMG) acquisition.

**Figure 3:**
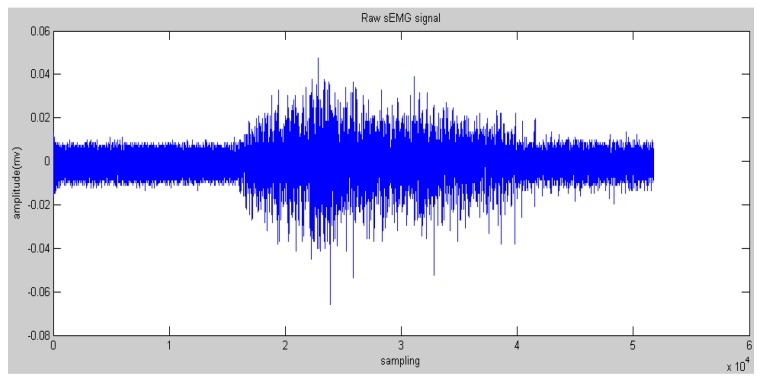
Example of raw sEMG signals recorded from bicep muscles.

The bipolar surface electrodes were used and the sampling rate is 1000 Hz (Figure 2). The electrodes are reusable and are adhered to bicep muscles, and a reference electrode is adhered to the body where no muscles exist as ground signals. The sampling data were pre-processed with a commercial sEMG acquisition and filter device (Oisaka Electronic Device Ltd., Fukuyama, Japan) with eight channels before sending to the processing program with the sampling rate of 1000 Hz. To obtain sufficient skin contact with the electrodes, every subject’s skin was shaved and cleaned with an alcohol swab. Figure 3 shows the recorded raw sEMG signals from a subject’s bicep muscle. The window length of the sEMG samples was set to 256 ms for the real-time requirement in an engineering application.

**Figure 4:**
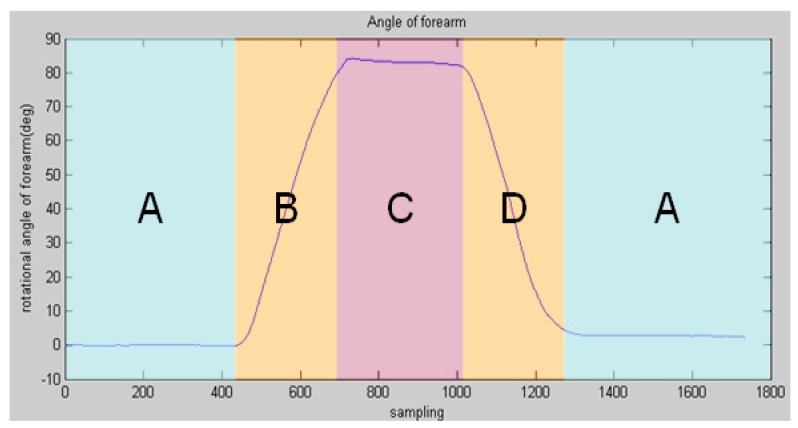
Motion of the subject’s forearm detected by an inertia sensor.

Sixteen subjects (healthy students in our lab) participated in the experiments. They were asked to perform elbow flexion and extension ten times as shown in Figure 1. The tilt angle of the forearm was detected via an inertia sensor when sEMG signals were being detected in Figure 4, which shows the four discrete patterns clearly. Because the frequency of sEMG acquisition is higher than that of angle value acquisition, the amount of sampling for angle information is less than that for sEMG signals.

### 2.2. Wavelet Packet Transform (WPT)

Wavelet transform was proposed because its advantages not only involve time and frequency domain features of EMG signals, but also exacting certain frequency domain features such as the frequency section of most EMG signals. Wavelet Packet Transform (WPT) generates a full wavelet basis decomposition tree. In each scale, not only the approximation signals, but also the detail signals are filtered to obtain two additional low and high-frequency signals.

For given sEMG signals *s*(*t*), whose scaling spaces are assumed as $U_{0}^{0}$, wavelet packet transform can decompose $U_{0}^{0}$ into small subspaces in a dichotomous way, which can be calculated according to Equation (1):

(1) $$U_{j+1}^{n}=U_{j}^{2n}⊕U_{j}^{2n+1},j\in Z;n\in Z_{+}$$

where j is the resolution level and ⊕ stands for orthogonal decomposition, $U_{j+1}^{n}$, $U_{j}^{2n}$ and $U_{j}^{2n+1}$ are three close spaces corresponding to $u_{n}(t)$, $u_{2n}(t)$ and $u_{2n+1}(t)$. $u_{n}(t)$ satisfies the following Equation (2) [14]:

(2) $$\{\begin{array}{c} u_{2n}(t)=\sqrt{2}\sum_{k\in Z}h(k)u_{n}(2t-k)\\ u_{2n+1}(t)=\sqrt{2}\sum_{k\in Z}g(k)u_{n}(2t-k) \end{array}$$

where the function $u_{0}(t)$ can be identified with the scaling function φ and $u_{1}(t)$ with the mother wavelet ψ. h(k) and g(k) are the coefficients of the low-pass and the high-pass filters, respectively. The sub-signals at $U_{j}^{n-1}$, and the *n*th subspace on the *j*th level, can be reconstructed by Equation (3):

(3) $$s_{j}^{n}(t)=\sum_{k}D_{k}^{j,n}\psi_{j,k}(t),k\in Z$$

where $\psi_{j,k}(t)$ is the wavelet function, and $D_{k}^{j,n}$ was the wavelet packet coefficients at $U_{j}^{n-1}$, which can be calculated by Equation (4):

(4) $$D_{k}^{j,n}=\int_{\infty }^{\infty }s(t)\psi_{j,k}(t)dt$$

**Figure 5:**
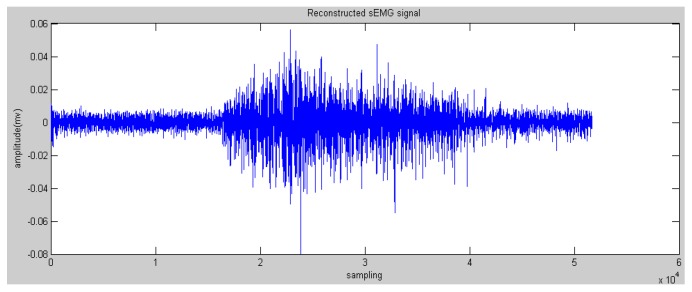
Reconstructed sEMG signals processed by Wavelet Packet Transform (WPT) in node 4.0.

**Figure 6:**
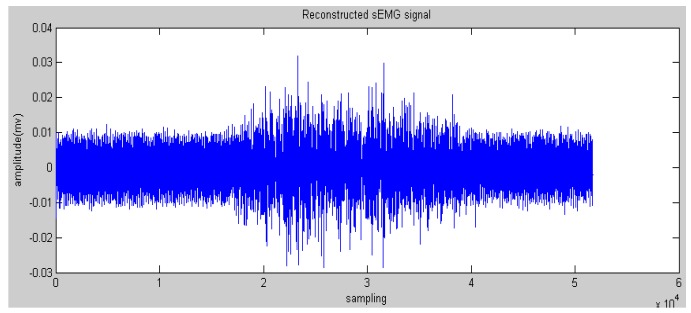
Reconstructed sEMG signals processed by WPT in node 4.1.

In this paper, the Daubechies wavelet was used as the mother wavelet. In addition, we chose Daubechies 2 and decomposed raw sEMG signals to the fourth level. The initial features can be obtained via the reconstructed wavelet via Equation (4). WPT generates a high-dimension feature vector. Some research proposed many methods to reduce the dimensions to save on calculation costs, such as principle component analysis and a self-organizing feature map [19]. In this paper, the most effective feature vectors are selected rather than all of the vectors, namely nodes 4.0 and node 4.1 (see Figure 5 and Figure 6) due to the effective components of sEMG signals being distributed in a low-frequency domain. This step could reserve the low-frequency signals and filter the high-frequency noise.

### 2.3. Feature Extraction Methods

After processing via WPT, sEMG signals were still not used to obtain the features of motion directly. The target of feature extraction is to obtain some typical data, which can reflect the motion patterns of human motion. Currently, most research in motion pattern recognition using sEMG signals focuses on discrete motion patterns such as hand opening, grasping, and so on, and traditional feature extraction methods can obtain the typical data to reflect the motion characteristics, such as MAV.

● Mean Absolute Value

(5) $$MAV=\frac{1}{N}\sum_{n=1}^{N}\left|s_{n}\right|$$

where $s_{n}$ is the reconstructed sEMG signals processed by WPT. The overlapped window is also used in this procedure.

**Figure 7:**
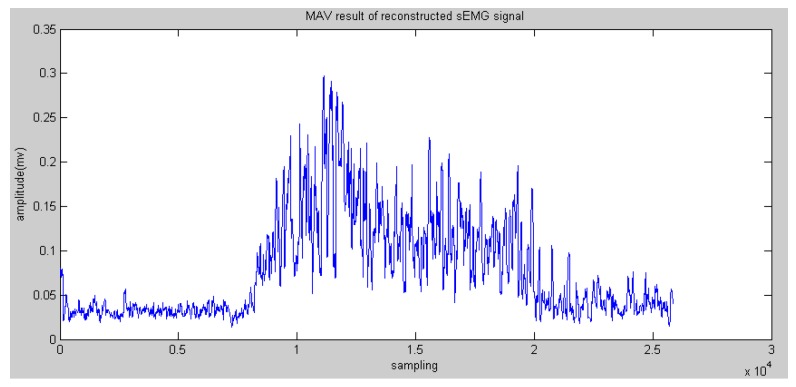
Mean Absolute Value (MAV) results of reconstructed sEMG signals in node 4.0.

**Figure 8:**
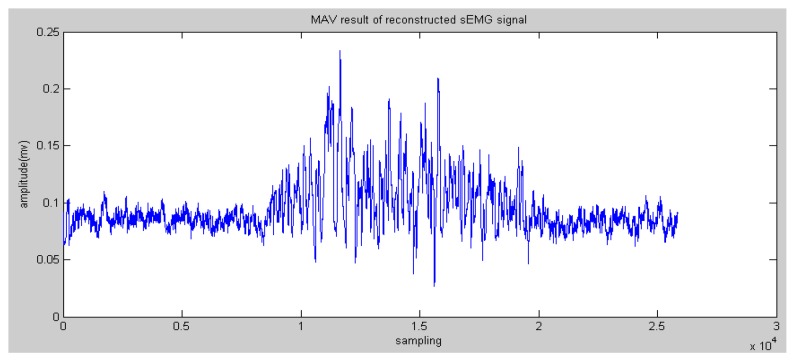
MAV results of reconstructed sEMG signals in node 4.1.

In general, the entropy of the reconstructed sEMG was commonly discussed. Meanwhile, Mahdi et al. [20] proved that static methods are also effective, such as MAV (see Figure 7 and Figure 8).

### 2.4. Weighted Peaks

In this research, motion patterns to be recognized are continuous in the time domain, which required the processed sEMG to have highly distinguishing characteristics related to elbow flexion and extension. To achieve this goal, a new feature extraction method was proposed in this paper with weighted peaks, which involve the following steps:

● Zero Crossing

(6) $$ZC=\sum_{n=1}^{N-1}\left[\mathrm{sgn}(s_{n}\times s_{n+1})\cap \left|s_{n}-s_{n+1}\right|\geq threshold\right]$$

where the $sgn\left(x\right)=\{\begin{array}{cc} 1 & \;\mathrm{if}\;x<0\\ 0 & \mathrm{otherwise} \end{array}$ threshold equals zero.

All the reconstructed sEMG signals of zero crossing are saved to obtain peaks and valleys among them.

● Trend Acquisition with Weighted Peaks

Human motion information is immersed in the peaks of sEMG signals, not in one peak, but in many series of peaks. Thus, groups of peaks are chosen and processed to find the implied information:

If $\max(s_{zc(i)}:s_{zc(i+1)})+\min(s_{zc(i)}:s_{zc(i+1)})\geq 0$

(7) $$P(i)=\max(s_{zc(i)}:s_{zc(i+1)})$$

**Figure 9:**
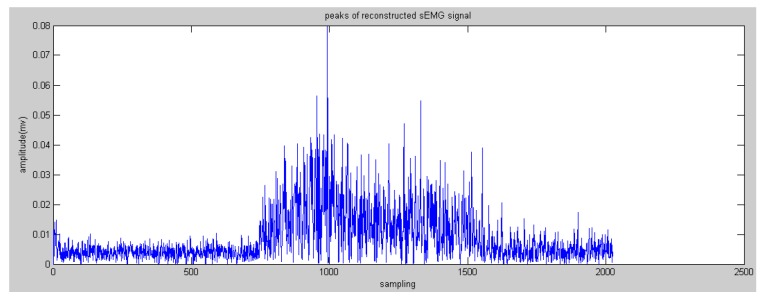
Peaks of the reconstructed sEMG in node 4.0.

**Figure 10:**
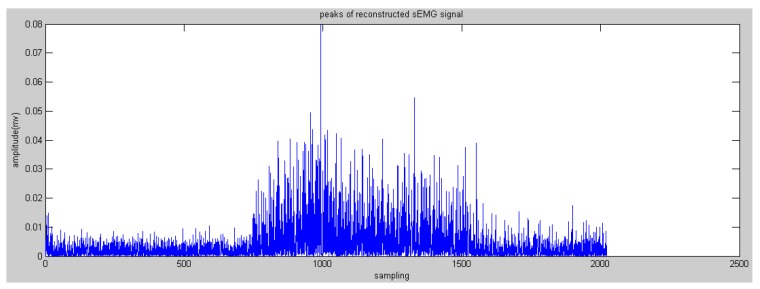
Peaks of the reconstructed sEMG in node 4.1.

Else if $\max(s_{zc(i)}:s_{zc(i+1)})+\min(s_{zc(i)}:s_{zc(i+1)})<0$

(8) $$P(i)=(-1)\times \min(s_{zc(i)}:s_{zc(i+1)})$$

where $s_{zc(i)}$ are the reconstructed sEMG signals of zero crossing; *P*(*i*) are the peaks or valleys between the data of zero crossing, and valleys are transformed into positive numbers (see Figure 9 and Figure 10).

**Figure 11:**
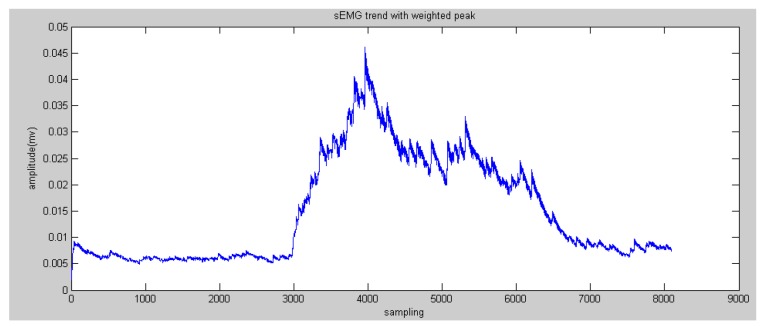
Results of weighted peaks of the reconstructed sEMG in node 4.0.

**Figure 12:**
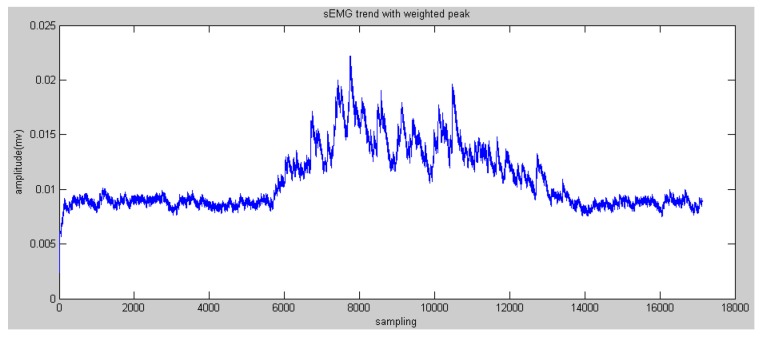
Trend acquisition from the reconstructed sEMG in node 4.1.

According to the comparison between the peaks of the reconstructed sEMG signals and human elbow flexion and extension, it was found that the higher peaks reflected the trend of motion more than the lower peaks. Therefore, the method of weighted peaks was proposed to increase the component of higher peaks and decrease the component of lower peaks to obtain features close to the motion of the subject’s forearm. Figure 11 and Figure 12 show the results of weighted peaks of the reconstructed sEMG in nodes 4.0 and 4.1, respectively. From the results of both figures, data of weighted peaks show a similar shape to that of the forearm angle trajectory. Therefore, it is possible to map these results of the forearm motion with the linear fitting method:

(9) $$P(i+1)=\frac{1-n}{n}P(i)+\frac{1}{n}P(i+1)$$

$$\{\begin{array}{cc} 3 & P(i+1)<P(i)\\ 1 & P(i+1)=P(i)\\ 1/3 & P(i+1)>P(i) \end{array}$$

● Adapted Weighted Peaks Method

From Figure 11 and Figure 12, the weighted peaks method aimed to extract features of the peaks of EMG signals based on increasing the component of the higher amplitude and decreasing the component of the lower amplitude. The weight bias of the high peaks can obtain the main features, however, the constant weighted parameter cannot smooth the feature trajectory because of the instability of the sEMG signals. Therefore, the variable weighted parameters will be useful to process the instable peaks. The main method to adapt the weighted peaks is tuning the weighted parameter based on the variation of current data to the previous data. If the value obtained by the weighted peaks method is a certain value B higher or lower than the previous value, then the method will be carried out once more, until the variation is under the certain value B. In fact, the effect of carrying out the weighted peaks method changed the weighted values.

If |P(i+1)−P(i)|>B,

(10) $$P(i+1)=\frac{1-n}{n}P(i)+\frac{1}{n}P(i+1)$$

If |P(i+1)−P(i)|≤B,

(11) P(i+1)=P(i+1)

### 2.5. Comparison of Features Extracted by the Proposed Methods and Traditional Methods

**Figure 13:**
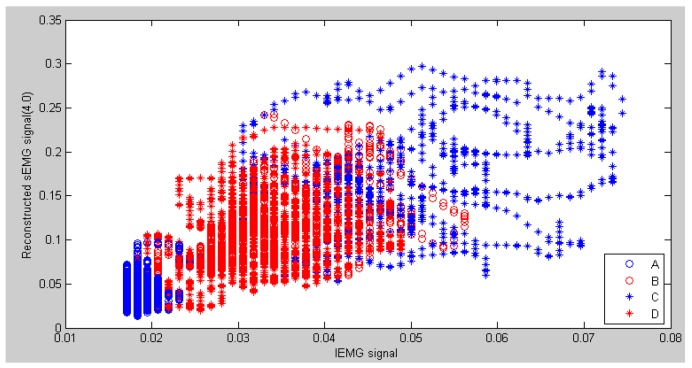
Example of a scatter figure on the integrated EMG (IEMG) signals and MAV of reconstructed sEMG signals in node 4.0.

Though traditional feature extraction methods perform well in discrete pattern recognition, they would not deal with the continuous pattern recognition using weak sEMG signals. In order to compare the proposed method of feature extraction via weighted peaks to obtain the sEMG trend with the traditional MAV method, LIBSVM library [21] developed by Chih-Chung Chang et.al was adopted to classify the four motion patterns mentioned previously. In each procedure of classification, the target vector can be generated automatically via the inertia sensor, and the feature vector is a 3 × n matrix. Figure 13 shows the scatter graph of distribution of the MAV results of reconstructed sEMG signals and integrated sEMG signals. From this figure, some features of patterns B and C overlapped in the adjacent area because of the strong fluctuation in MAV results, which can reduce the recognition rate. On the other hand, patterns B and D almost overlap each other, which cannot be recognized directly. The reason is that discrete features can only represent the magnitude of muscle activation and not reflect the direction of motion. It could be solved by using the previous status and obtaining the current status.

**Figure 14:**
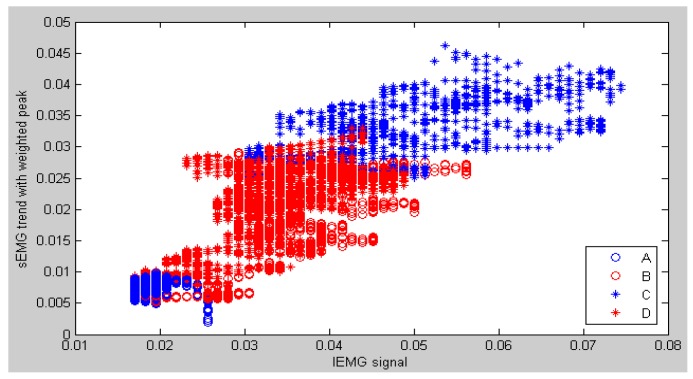
Example of a scatter figure on IEMG signals and a sEMG trend with weighted peaks in node 4.0.

Figure 14 shows the scatter graph of an sEMG trend with a weighted peak and the IEMG signals. From this figure, the superposition area between pattern B and pattern C obviously decreases because the variation trend of sEMG signals is smooth and reflects the motion of the forearm. It is also obvious that the sEMG trend with weighted peaks has more discriminative features than MAV features. The results of whole contrastive experiments are shown in Table 1. From this table, the recognition rate of weighted peaks is higher than that in the MAV for all of the subjects.

### 2.6. Linear Map from sEMG Signals to Forearm Motion

Because each subject was required to perform elbow flexion and extension with his upper arm relaxed in the sagittal plane at a very low and constant speed, the dynamics of the forearm can be omitted. The processed sEMG signals derived via weighted peaks and adapted weight peaks show a trend of signal variation consistent with the human motion at a certain degree; further details are given in the following section. Therefore, it is possible to use simple linear fitting to build the relationship between the sEMG signals and elbow flexion and extension. Namely, the activation of the bicep muscle is considered to be proportional to the motion of the subject’s forearm. On the other hand, to implement the real-time continuous recognition, we adapted a simple proportional fitting line to map the weighted peaks of sEMG signals to the motion:

(12) M(i)=aP(i)+b

The least squares method was used to obtain the parameters *a* and *b*. For example, according to the ten-time experimental results from subject 1, the average of *a* is 710, and the value of *b* is −43.

## 3. Experiments and Results

**Figure 15:**
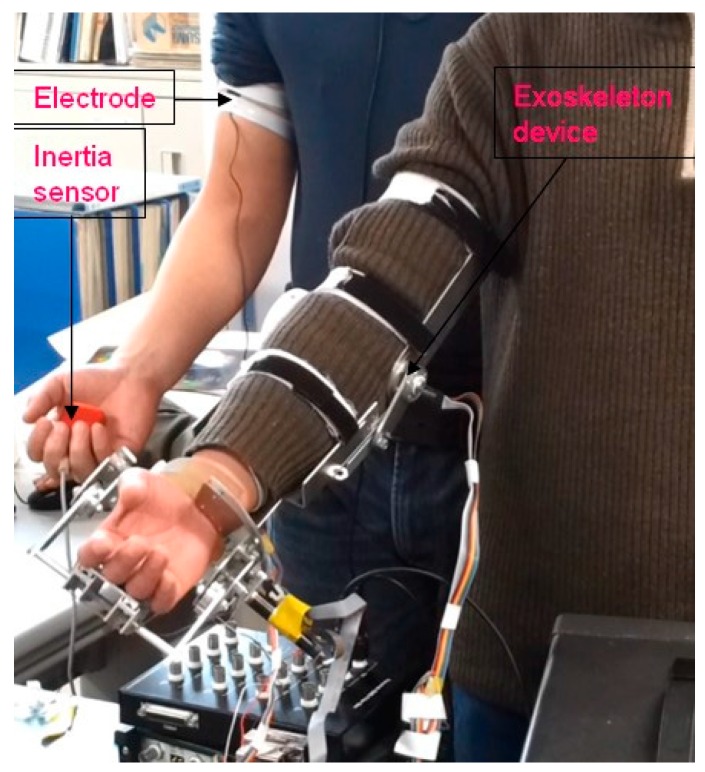
Two subjects performing elbow flexion and extension, where one subject attached one electrode to the surface of the bicep muscle, and the other subject wore an exoskeleton device.

Four of the subjects were required to perform elbow flexion and extension in the sagittal plane with their upper arms relaxed. The rational angle of the forearm is about 90 deg in the sagittal plane from a posture with the forearm vertical to the ground to the posture of the forearm being parallel to the horizontal plane. Figure 15 shows that one subject was grasping an inertial sensor and one electrode was attached to his bicep muscle, and the other subject wore an exoskeleton device. All of the subjects were asked to acquire the sEMG signals from their bicep muscles, and one subject wore an exoskeleton device to evaluate the efficacy of the proposed methods. The experiment includes two levels. In the first level, the posture of the subject’s forearm was predicted through the weighted peaks method. In this level, elbow flexion and extension were required to be done three times in one trial. Each subject was required to perform elbow flexion and extension ten times. After one trial was done, the subject was allowed to rest for one minute. In the second level, subjects were asked to wear the device on one upper limb where the electrode was attached. The improved weighted peaks were used to process the sEMG signals. Just like the first level, the experiments were required to be performed ten times.

**Figure 16:**
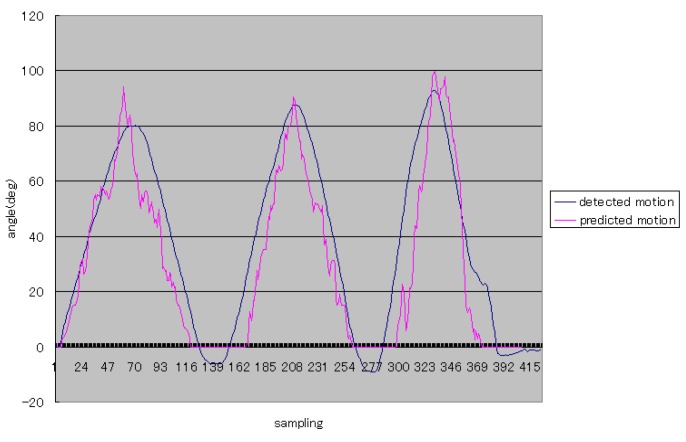
The typical elbow flexion and extension for Subject 1.

One example of the first level of experiments was shown in Figure 16. In this figure, the vertical axis stands for the angle of the forearm, and it is easy to understand that Subject 1 performed elbow flexion and extension three times during status A and status B (shown in Figure 1). The blue curve means the rational angle of the forearm detected by using the inertia sensor. The red curve stands for the rational angle obtained by using the proposed method. Because the subjects perform the elbow flexion and extension from 0 deg to 90 deg, we did the simple processing that limits the minimum values of predicted results.

**Figure 17:**
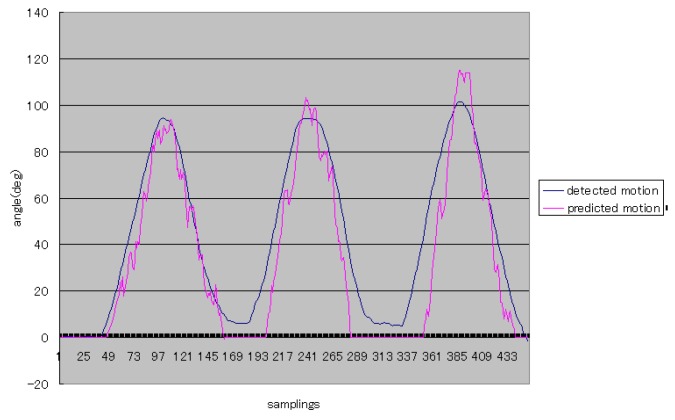
The typical elbow flexion and extension for S2.

Figure 17 shows more typical experimental results from Subject 2, who obtained better prediction for continuous posture of elbow flexion and extension during the first time. On the other two occasions, the performance did not obtain good prediction due to the unstable sEMG signals. It is also indicated that it is difficult to predict continuous posture for upper limbs with weak sEMG signals.

**Figure 18:**
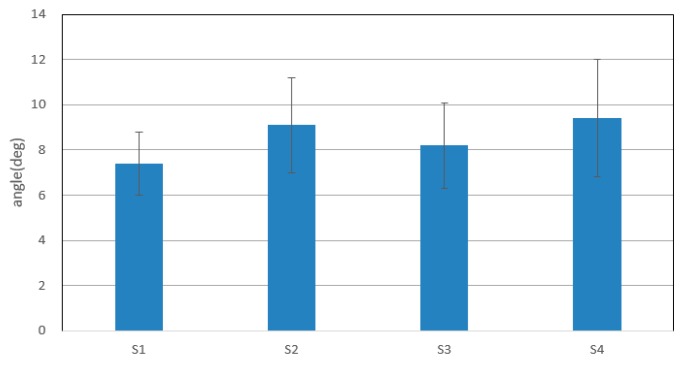
Average errors between detected motion and predicted motion in the first level experiment.

Figure 18 shows the average errors between elbow flexion and extension detected via the inertia sensor and predicted angles of four subjects for ten experiments. Subject 1 shows the best prediction results among them, of less than 8 deg. Other subjects also represented acceptable results of continuous recognition of elbow flexion and extension.

**Figure 19:**
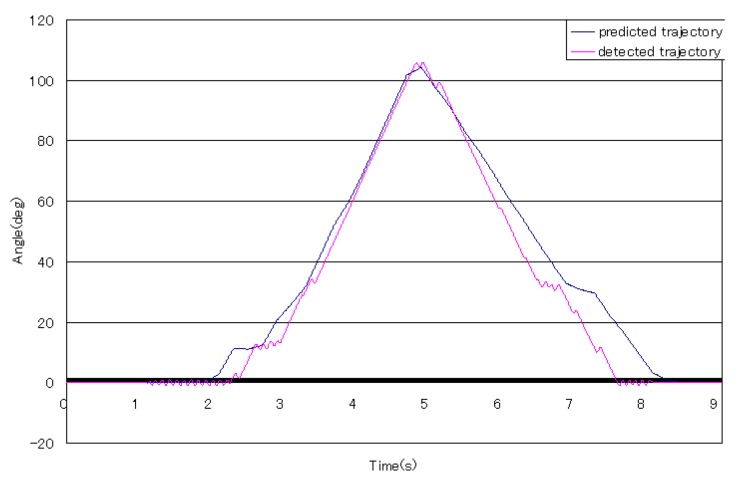
The typical elbow flexion and extension for S1.

**Figure 20:**
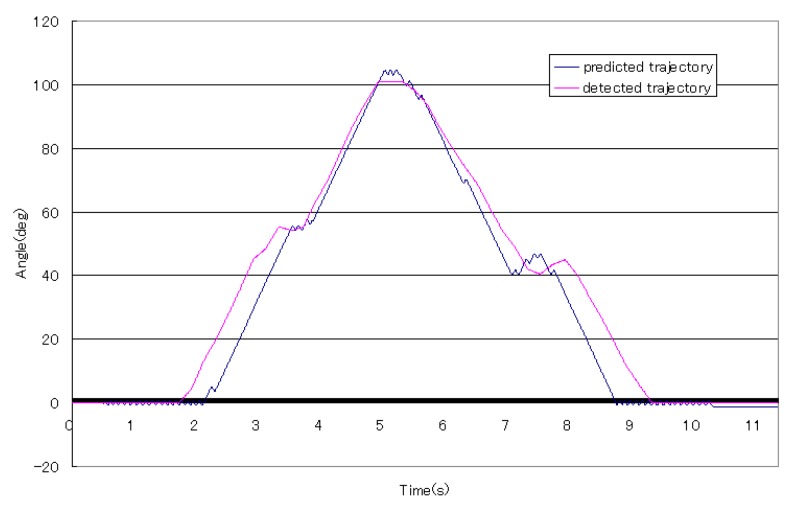
The typical elbow flexion and extension for S2.

From Figure 16 and Figure 17, the trajectories of predicted angles of the forearm are not smooth, but the trajectories of detected angles of the forearm are smooth; caused by the ability of motion trend identification of the proposed weighted peaks. Thus, in the following experiments, adapted weighted peaks were used to process the sEMG signals, which are shown in Figure 19. In this figure, the trajectories of prediction and detection for elbow flexion and extension in the sagittal plane are almost the same. The predicted trajectory is more accurate than that in the first level experiment, which indicated that the improved weighted peaks method is more effective in reducing the instability of the sEMG signals. Though the trajectory is more accurate than that obtained by the weighted peaks method, there is still some instability where the variation of data is low because the certain value *B* was not set low enough. However, if the certain value *B* was set too low, the variation of sEMG peaks would be reduced, and the posture of the subjects’ forearms could not be predicted accurately. Figure 20 shows the S2’s elbow flexion and extension, which are obtained through prediction and detection in the second level experiment. This result also indicated that the predicted trajectory is almost the same as the detected trajectory.

**Figure 21:**
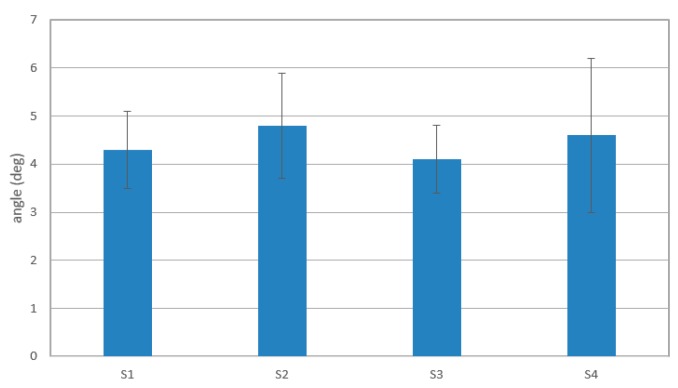
Average errors between detected motion and predicted motion in the second level experiment.

Figure 21 shows the average errors between detection and prediction motion of four subjects using the adapted weighted peaks method to process the sEMG signals. In this figure, the errors are lower than those in the experiments using the weighted peaks method. Different from the results in the first level experiments, the S3 obtained the smallest errors. Another difference between the second level results and the first level results is the smoothness of the angle trajectory and processed sEMG signals, which was induced by the different iteration times of both levels, with the second level requiring more iterations. Thus, the results of angle trajectory and sEMG signal trend are less smooth than those in the first level.

## 4. Discussion

With the increasing demands of rehabilitation for patients who have lost motor function, preliminary research for bilateral rehabilitation based sEMG signals was conducted in this paper, where human elbow flexion and extension were recognized continuously with only sEMG signals from biceps used during the experiments conducted. Wavelet transform packet is used to filter the raw sEMG signals. To obtain the variation trends of sEMG signals which are relative to human motion, a new method of processing of weighted peaks of sEMG signals is proposed as well as adapted weighted peaks. Moreover, the linear fitting method was used to map the features calculated by the proposed methods to the motion of elbow flexion and extension, so that the continuous motion of the forearm in the sagittal plane can be obtained within an acceptable range. According to the contrastive experiments, the proposed methods obtained the trend of sEMG signals with a higher recognition rate for both subjects.

## 5. Conclusions

SEMG signals have the potential to be used to estimate the dynamics of human limbs, which is of benefit to rehabilitation, especially to bilateral rehabilitation, where hemiplegic patients can train their affected limbs following the motion of unaffected limbs via some rehabilitation devices. Traditional methods to process the sEMG focused on motion discrete pattern recognition, which is not satisfactory for use in bilateral rehabilitation. To overcome this problem, in this paper, the mapping between sEMG signals and human motion in elbow flexion and extension on the sagittal plane was proposed. Participants were required to perform elbow flexion and extension on the sagittal plane smoothly, with only an inertia sensor in their hands, without considering forearm dynamics, where sEMG signals were weak compared to those with heavy loads or high acceleration. The contrastive experimental results show that continuous motion can also be obtained within an acceptable precision range. In the future, these results will be improved and used to develop the exoskeleton device in order to aid bilateral rehabilitation for upper limbs and lower limbs.

## Figures and Tables

**Table 1:** Average recognition rate of contrastive experimental results (%).

	MAV	Weighted Peaks
Subject 1	85.1	93.4
Subject 2	86.6	95.3
Subject 3	79.3	87.7
Subject 4	81.1	89.3
Subject 5	81.7	91.9
Subject 6	80.3	87.0
Subject 7	77.8	84.1
Subject 8	79.0	90.6
Subject 9	80.3	89.1
Subject 10	85.7	89.9
Subject 11	81.8	92.7
Subject 12	82.1	91.5
Subject 13	80.3	93.5
Subject 14	77.1	90.7
Subject 15	79.1	85.1
Subject 16	81.9	89.2

## Abbreviations

SEMG	Surface Electromyography
WPT	Wavelet Packet Transform
IEMG	Integrated Electromyography
MAV	Mean Absolute Value
